# The Genus *Cynometra*: A Review of Ethnomedicine, Chemical, and Biological Data

**DOI:** 10.3390/plants11243504

**Published:** 2022-12-14

**Authors:** Shabnam Sabiha, Rita Serrano, Kamrul Hasan, Isabel B. Moreira da Silva, João Rocha, Nurul Islam, Olga Silva

**Affiliations:** 1 Research Institute for Medicines (iMed.ULisboa), Faculty of Pharmacy, Universidade de Lisboa, 1649-003 Lisbon, Portugal; 2 Department of Zoology, Faculty of Biological Sciences, University of Rajshahi, Rajshahi 6205, Bangladesh

**Keywords:** antimicrobial, anti-inflammatory, *Cynometra*, cytotoxic, herbal medicines, secondary metabolites, traditional medicine

## Abstract

*Cynometra* L. is a *Fabaceae* genus that is widely distributed throughout the tropics, consisting of tropical forest trees with ecological and economic importance since they are used as food and herbal medicines by the populations of their natural habitats. Our goal is to provide a review of the research data concerning the potential of this botanical genus as a source of herbal medicines and secondary metabolites that are useful for human health. To that end, scientific databases, including PubMed, Science Direct, ISI Web of Science, Scopus, and Google Scholar, were searched using the following terms: *Cynometra*, medicine, chemical, biological activity, toxicity, and “AND” as the Boolean connector. Eleven *Cynometra* species (9.7%) were reported to be used in traditional medicine to treat different ailments. A total of 185 secondary metabolites of various chemical classes, mainly flavonoids and terpenoids, were identified in eight *Cynometra* species (7.1%). Vitexin was the only flavonoid identified as bioactive in the sequence of bioguided studies on this botanical genus. Ten species (8.8%) were submitted to in vitro and in vivo biological activity assays. The main evaluated activities were in vitro antioxidant, antimicrobial, cytotoxic, and in vivo anti-inflammatory activities, but no human clinical trials or safety data about this genus were found. *Cynometra cauliflora* and *Cynometra ramiflora* were the most studied species. The present work confirms the use of *Cynometra* species as a source of medicinal plants. However, more experimental studies must be conducted to better understand this botanical genus’s usefulness as a source of raw materials for pharmaceutical use.

## 1. Introduction

The genus *Cynometra* L. is a species-rich genera in the most significant tropical family *Fabaceae* (*Leguminosae*) and subfamily *Detarioideae*, described for the first time in 1741 by Linnaeus and included in the first edition of *Species Plantarum* (published in 1753) [1]. This botanical genus has a wide distribution and high diversity. It is classified using regional groupings of species (the Neotropics, Tropical Africa, Madagascar, the Comoros Islands, and the Indo-Pacific groups) [1,2,3]. According to phylogenetic studies, the *Cynometra* genus is polyphyletic [4,5,6,7,8].

According to the data of Plants of the World Online (https://powo.science.kew.org/; accessed on 5 November 2021) [9], The plant list (http://www.theplantlist.org/; accessed on 1 June 2021) [10], the World Flora Online (WFO) (http://www.worldfloraonline.org./; accessed on 1 January 2022) [11], and the Global Biodiversity Information Facility (GBIF) (https://www.gbif.org/; accessed on 1 January 2022) [12], the genus *Cynometra* integrates 113 species (Table 1) of shrubs to large trees. It has a broad tropical distribution [9]. They grow in tropical lowland, rain, and swamp forests, often along rivers and sublittoral zones, and seasonally also in dry forest, woodland, bushland, or thickets, often on white sands. Some species grow gregariously, forming dominant stands; some are prominent mangrove species. [9,13]. 

**Figure 1:**
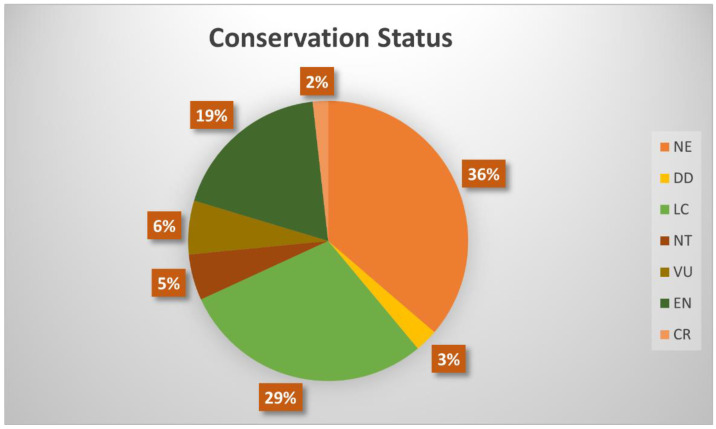
Conservation status of *Cynometra* species. NE—not evaluated, DD—data deficient, LC—least concern, NT—near threatened, VU—vulnerable, EN—endangered, CR—critically endangered.

Concerning the status of *Cynometra* species and based on the Red List of Threatened Species of the International Union for the Conservation of Nature (IUCN) [14], 36% of these species are considered as not evaluated (NE), 29% as least concern (LC), 19% as endangered (EN), 6% as vulnerable (VU), and 5% as near threatened (NT). Additionally, 3% and 2% are referred to as data deficient (DD) and critically endangered (CR), respectively (Figure 1). 

*Cynometra* species are generally recognized as used in traditional medicine in the countries where they exist as part of the spontaneous flora. Traditional practitioners usually prepare medicine from different plant parts and by different modes of preparation to treat various ailments. However, it should be noted that only a little information was available related to the concrete use for the treatment of different pathological signals or symptoms and their chemical, pharmacological, and toxicological properties. So, to gain and give a clear idea about a genus, it is very important to collect, arrange, and review all necessary information concerning medicinal importance of the genus. 

In the present work, a revision of the ethnomedical, chemical, pharmacological, and toxicological data on the genus *Cynometra* is presented and discussed to better characterize the potential of this botanical genus as a source of medicinal plants and traditional herbal medicines, and as a source of natural products that could be useful for the development of new drugs. 

## 2. Results and Discussions

### 2.1. Selection of the Information

**Figure 2:**
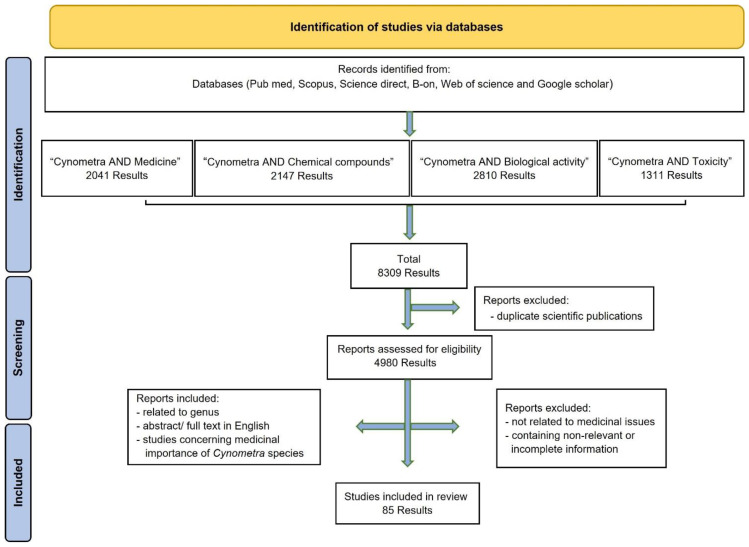
Data screening based on PRISMA methodology.

**Figure 3:**
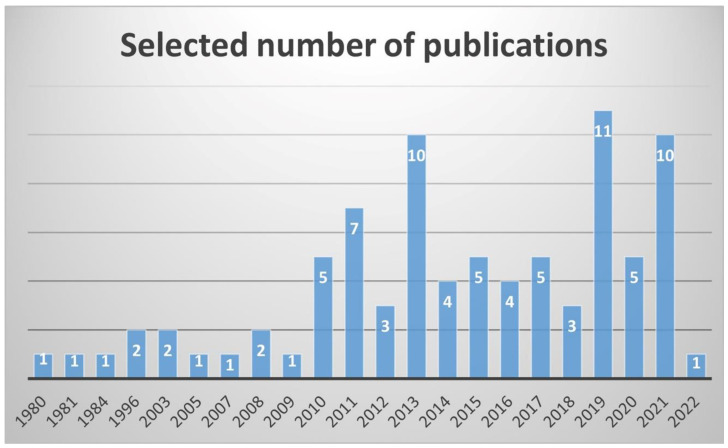
Number of selected *Cynometra* scientific publications by year.

Details of data collection and choice are given in Figure 2. The initial titles and abstract search yielded 8309 results. After excluding duplicates, 4980 scientific publications were reviewed for eligibility. Of those, 4895 scientific publications were excluded for the following reasons: repeated results, no relation to medicinal issues, and inclusion of irrelevant or incomplete information. Finally, a total of 85 scientific publications were considered eligible to be included in this review. The inclusion criteria were publications related to *Cynometra* genus; abstracts or full texts in English; and studies on *Cynometra* species concerning medicinal importance. In Figure 3, the number of selected scientific publications according to the respective publication years is presented. 

### 2.2. Ethnomedicinal Data

Eleven *Cynometra* species, i.e., *C. brachyrrhachis*, *C. capuronii*, *C. cauliflora*, *C. hankei*, *C. iripa*, *C. manii*, *C. megalophylla*, *C. ramiflora*, *C. spruceana*, *C. vogelii*, and *C. webberi*, have been reported for their ethnomedicinal uses (Table 2). The leaf, fruit, seed, stem, bark, resin, and root of these species are traditionally used for the treatment of digestive disorders, respiratory problems, skin, and inflammatory diseases. For example, the decoction of the *C. cauliflora* leaf is used to treat diabetes and hyperlipidemia [15]; however, in Indonesia, the fruit of this species is used as food, and the leaf is used as medicine for the treatment of diarrhea [16]. 

The leaf was found to be the most used *Cynometra* plant part for medicinal purposes. The decoction and powder are mainly used in the preparation of herbal medicine. 

According to our results, among the total number of 113 *Cynometra* species, only 9.7% have been recorded for their traditional uses (Table 2).

### 2.3. Chemical Compounds

In Table 3, the main compounds isolated and identified from the studied chemicals of eight *Cynometra* species (7.1%) are presented. Flavonoids and terpenoids are the major chemical classes reported on this botanical genus beside fatty acids, alkaloids, esters, and other phenol derivatives. *C. cauliflora* was the most studied plant species. 

The presence of tannins, flavonoids, and terpenoids was reported in the aqueous extracts of stem, bark, and leaf [19], as well as in a methanolic extract of *C. cauliflora* leaf [33]. Cardiac glycosides were present in the different parts of the plant, except on the stem [19]. Ethanol extract of *C. ramiflora* leaf was revealed to contain alkaloids, phenolic compounds, and terpenoids (saponins and steroids) [34,35]. The existence of tannins was found in the ethanol, hexane, and dichloromethane extracts of the stem and root of *C. vogelli* [32]. Preliminary phytochemical screening of an ethanolic extract of *C. malaccensis* leaf, twig, and stem bark showed the presence of flavonoids, terpenoids, and high content of tannins. [36]. The presence of alkaloids in the leaf and stem [37] and different type of fatty acids in leaf and seed [38] have been reported in *C. iripa*. Basak et al. (1996) also noted the presence of chlorophyll, carotenoids, proteins, polyphenols, and tannins in the seed of this species [39]. The existence of phenol derivatives, including gallotannins, leucoanthocyanins and anthraquinones, and of saponins and steroids were reported from *C. capuronii* leaf [18].

In the essential oil of *C. cauliflora* leaf, twig, and fruit twenty-six, seventeen, and fifty compounds (mainly monoterpenoids and sesquiterpenoids) were identified, respectively. For the leaf oil, the major constituents were *α*-terpineol (34.62%), (*z*)-*β*-ocimene (20.77%), and *γ*-terpinene (12.27%); meanwhile, trans-sabinene hydrate (58.77%), an oxygenated monoterpenoid, dominated the twig oil. On the other hand, oxygenated sesquiterpenoids were predominant in the fruit essential oil, accounting for 65.48% of the total essential oil content [40]. Different flavonoids such as apigenin, xanthotoxin, catechin, cyanidin, and vitexin [15,41], have also been identified in the leaf of this species (Table 3).

From the *C. megalophylla* root essential oil, 43 compounds were identified. Monoterpenoids were the major constituents of it, namely, *α*-phellandrene (32.0%), *p*-cymene (18.2%), and *γ*-terpinene (12.1%) [42]. 

Gartlan et al. (1980) reported the presence of cyanidin in the mature leaf and seed of *C. hankei* [43]. 

At least 14 fatty acids were found in the oil of *C. iripa* seed, while 10 fatty acids were in the leaf oil. Linoleic acid (34.2%) was prominent in seed oil, and palmitic acid (33.5 %) was prominent in leaf oil [38]. 

**Figure 4:**
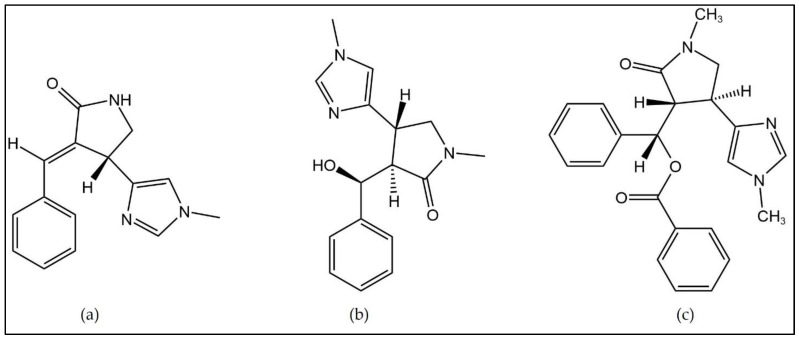
Examples of Imidazole alkaloids (**a**) anantine, (**b**) cynometrine, and (**c**) cynodine identified on *Cynometra* species.

The presence of imidazole alkaloids that are characteristic of this botanical genus were noticed in *C. anata* (leaf) [44], *C. hankei* (stem bark and seed) [45], and *C. lujae* (not indicated) [44]. In Figure 4, some examples of imidazole alkaloids are given. 

Some chemical studies related to the quantification of representative secondary metabolites classes were also performed. The total phenolic content (TPC) of a young leaf of *C. cauliflora* was found to be 1831.47 ± 1.03 mg GAE (gallic acid equivalent)/g, and the total flavonoid content (TFC) was found to be 33.63 ± 0.25 mg CE (catechin equivalent)/g [19]. However, the ethanol extract of the leaf and fruit of *C. cauliflora* was reported to have TPC 344.17 ± 10.80 and TPC 122.04 ± 3.17 mg GAE/g plant extract [46,47]. The methanol and aqueous extracts of *C. cauliflora* fruit showed a TPC of 1868.94 ± 11.68 (mg GAE/100 g edible portion) and of 1.30 ± 0.10 (mg GAE/g dry weight), respectively, [48,49], whereas in another study, the aqueous extract of this species showed TPC 4.6 ± 0.06 mg GAE/g dry weight. The TMAC (total monomeric anthocyanin content) and vitamin C content of *C. cauliflora* fruit aqueous extract were 8.66 ± 1.68 and 21.8 ± 0.33, respectively [50]. In a recent study, Abeysuriya et al. (2020) reported low content of vitamin C (37.9 ± 1.8 mg/100 g fresh weight) from seedless fruit extract of *C. cauliflora* (extraction solvent: 3% (*w*/*v*) meta-phosphoric acid and 8% (*v*/*v*) glacial acetic acid) and medium TPC (428.5 ± 1.3 mg GAE/100 g fresh weight) and TFC (26.1 ± 1.0 mg QE (quercetin equivalent)/100 g fresh weight) from MeOH (methanol) extract of the same [51]. 

In a methanol extract of *C. ramiflora* stem, TPC, TFC, and total tannins content were found to be 96.2 mg GAE/g, 166.4 mg QE/g, and 80.4 mg GAE/g dry weight, respectively [52].

**Table 3:** Chemical compounds identified from *Cynometra* species.

Species	Part Used	Chemical Class	Compounds	Ref.
*C. anata*	leaf	Alkaloids	anantine, cynometrine and cynodine	[44]
*C. cauliflora*	leaf	Flavonoids	xanthotoxin, fraxetin, capensine, naringenin, malvidin, cyanidin, amorphigenin, nobiletin, isorhamnetin, epigallocatechin, gallate, apigenin, and oenin	[41]
	stem	Flavonoids	apigenin	[53]
	twig	Flavonoids	naringenin, eriodictyol, apigenin, acacetin, luteolin, luteolin 3’,5 dimethyl ether, 3’,4’,7-trihydroxyflavone, 4’,7-dihydroxyflavone and 5,7-dihydroxychromone	[54]
	leaf	Mono- terpenoids	*α*-thujene, *α*-pinene, *β*-pinene, myrcene, *δ*-3-carene, *α*-terpinene, *p*-cymene, limonene, (*z*)-*β*-ocimene, *γ*-terpinene, terpinolene, linalool, *α*-terpineol, *neo*-dihydrocarveol, *cis*-carvone oxide, trans-dihydro-a-terpinyl acetate	[40]
		Sesqui- terpenoids	*α*-bulnesene, *β*-chamigrene, *α*-himachalene, *trans*-cadin-1,4-diene	
		Phenols	*p*-vinyl guaiacol	
		Hydrocarbons	(3*E*)-2-methyl-octen-5-yne	
	twig	Mono- terpenoids	(*z*)-*β*-ocimene, santolina alcohol, (*E*)-*cis*-jasmonol, *cis*-verbenol, linalool, geraniol, *cis*-4-caranone, *trans*-sabinene hydrate, dihydromyrcenol	
		Sesqui- terpenoids	squamulosone, occidol acetate, *α*-eudesmol acetate	
		Fatty acids	octanoic acid, decanoic acid, dodecanoic acid, linoleic acid	
		Flavonoids	fragranol	
	fruit	Sesqui- terpenoids	*β*-cubebene, *β*-elemene, *α*-guaiene, prezizaene, ishwarane, *β*-chamigrene, germacrene d, *α*-muurolene, *β*-bisabolene, *α*-bulnesene, *γ*-cadinene, (*E*)-*γ*-bisabolene, *γ*-cuprenene, *trans*-cadina-1,4-diene, selina-3,7(11)-diene, 9-epi-(*E*)-caryophyllene, *α*-chenopodiol, longiborneol, trans-*β*-elemenone, *α*-acorenol, agarospirol, occidenol, cryptomerione, curcumenol, hinesol, nootkatol, sesquisabinene, *α*-muurolol, *β*-calacorene, *γ*-eudesmol, elemol, eremoligenol, (2*E*,6*E*)- farnesol, (*E*)-nuciferol, (*z*)-lanceol, 11-*αH*-himachal-4-en-1-*β*-ol, globulol, cubebol, longipinanol, valerianol, allohimachalol, epi-*β*-bisabolol, occidol acetate, longiborneol acetate	
		Mono- terpenoids	limonene, *cis*-thujone, *trans*-pulegol, *cis*-*β*-farnesene	
		Fatty acids	linoleic acid	
	leaf	Condensed tannins	procyanidin trimer, procyanidin tetramer, procyanidin hexamer	[15]
		Flavonoids	catechin, taxifolin pentoside, vitexin, isovitexin, kaempferol hexoside, quercetin pentoside, quercetin hexoside, apigenin-6-*C*-glucoside-8-*C*-glucoside, kaempferol–coumaroyl hexoside and isorhamnetin hexoside	
*C. hankei*	Leaf, seed	Flavonoids	cyanidin	[43]
	stem bark, seed	Alkaloids	*N*_1_-demethyl cynometrine, *N*_1_-demethyl cynodine, cynometrine, and cynodine	[45]
*C. iripa*	leaf, seed oil	Fatty acids	leaf—lauric acid, myristic acid, pentadecanoic acid, palmitic acid, stearic acid, arachidic acid, behenic acid, oleic acid and *cis*-11-eicosenoic acid, linolenic acidseed—caproic acid, lauric acid, myristic acid, palmitic acid, stearic acid, arachidic acid, behenic acid, tricosanoic acid, lignoceric acid, oleic acid, linoleic acid, linolenic acid, *cis*-8, 11, 14-eicosatrienoic acid, *cis*-13, 16-docosadienoic acid	[38]
	seed, seed coat	Triterpenoids	squalene, *β*-sitosterol, stigmast-4-en-3-one, cholesta-4,6-diene-3-ol (3-*beta*)	[55]
		Tetraterpenoids	*β*-carotene	
		Esters	1,2-benzenedicarboxylicacid mono (2-ethylexyl) ester, butyric acid 2-pentadecyl ester, 1,2-benzenedicarboxylic acid butyl 2-ethylhexyl ester	
		Fatty alcohols	1-eicosanol, falcarinol	
		Quinones	2,5-di-*ter*-butyl-1,4-benzoquinone	
		Phenolic aldehydes	3,5-di-*ter*-butyl-4-hydroxybenzaldehyde	
		Vitamins	vitamin E	
		Hormones	progesterone	
*C. lujae*	not indicated	Alkaloids	anantine, cynometrine, isoanantine, isocynometrine, isocynodine noranantine, hydroxyanantine and cynolujine	[44]
*C. megallophylla*	root oil	Mono- terpenoids	*α*-pinene, α-thujane, sabinene, *β*-pinene, myrcene, *α*-phellandrene, *α*-terpinene, *p*-cymene, limonene, *β*-phellandrene, (*E*)-*β*-ocimene, *γ*-terpinene, terpinolene, 1,8-cineole, *cis*-p-menth-2-en-1-ol, *trans*-*p*-menth-2-en-1-ol, borneol, terpinen-4-ol, carvacrol, *p* -cymen-8-ol, *trans*-piperitol, terpinen-4-yl acetate	[42]
		Sesqui- terpenoids	caryophyllene oxide,* α*-eudesmol,* β*-eudesmol, hinesol, globulol, *β*-selinene, germacrene *D*, allo-aromadendrene, *α*-humulene, *α*-guaiene, *β*-caryophyllene	
		Hydrocarbon	decane, dodecane, undecane, *N*-tridecane, tetradecane, pentadecane, hexadecane, heptadecane, octadecane	
*C. ramiflora*	leaf	Triterpenoids	glutinol, glutinone, *β*-sitosterol	[56]
		Ester	ethyl 4- ethoxy benzoate	
*C. vogelii*	leaf oil	Sesquiterpenoids	*β*-caryophyllene, *α*- and *β*-selinene	[57]
		Fatty acid	isopropyl palmitate	

### 2.4. Biological Studies 

Results of the in vitro and in vivo biological activity tests made on the *Cynometra* genus are summarized in Table S1 (please consult our supplementary data, all references are orderly according to its occurrence on this table). A total of ten species (8.8%), namely, *C. bauhiniifolia*, *C. brachyrrhachis*, *C. cauliflora*, *C. cloiselii*, *C. iripa*, *C. madagascariensis*, *C. ramiflora*, *C. spruceana*, *C. travancorica*, and *C. vogelii*, were studied. Among them, *C. cauliflora* and *C. ramiflora* were found to be the most important species concerning biological activities. Methanol and ethanol were mostly used as extraction solvents, and leaf and fruit were the most important plant parts to show different biological activities. 

The leaf and fruit of *C. cauliflora* were the most biologically tested plant parts of this species:

A methanol leaf extract showed significant antioxidant [15,60,62,63]; antibacterial (against *Staphylococcus aureus*, *Escherichia coli*, *Porphyromonas gingivalis*, and methicillin-resistant *Staphylococcus aureus* [33,62,67]); anti-viral (against herpes simplex virus type 1) [71]; anti-diabetic; antidiarrheal (in vivo) [63]; and cytotoxic potentiality against brine shrimp (*Artemia salina*) and Vero cells [71,72]. An ethanol leaf extract showed anti-inflammatory activity by inhibiting the activities of arachidonate-5-lipoxygenase and hyaluronidase [64]. In addition, the same extract exhibited strong antioxidant and high inhibitory alpha-glucosidase activities [46], as well as moderate cytotoxic activity (against HeLa cancer cells) [74]. Moreover, this extract and vitexin, a flavonoid isolated from this medicinal plant, were observed to be involved in its in vivo anti-obesity and lipid-lowering activities [69], and an aqueous leaf extract showed antioxidant and potent anti-diabetic activity in vivo [65,73].

A methanol extract of the fruit exhibited cytotoxic activity (against human promyelocytic leukemia HL-60 and normal mouse fibroblast NIH/3T3 cell lines) and low antioxidant activities [46,59]. A fruit’s hexane, chloroform, ethyl acetate, ethanol, methanol, and aqueous extracts showed antifungal activity against four species of yeasts (*Candida albicans*, *Candida parapsilosis*, *Candida krusei*, and *Cryptococcus neoformans*), and two species of filamentous fungi (*Aspergillus fumigatus* and *Trichophyton interdigitale*) [66]. In two other studies, the fruit aqueous extract showed significant antioxidant activity [49,50]. In contrast, the methanol extract of the same plant part showed a low antioxidant activity [48].

Concerning *C. cauliflora*, the stem and the essential oils were also studied:

The stem ethyl acetate and methanol extracts showed, strong antioxidant and anti-cholinesterase activities (>80% inhibition), respectively [53,68].

The essential oils obtained from leaves, twigs, and fruits showed antioxidant activity, whereas the observed twig oil was more active than the oil from the other plant parts and showed significant antibacterial and cytotoxic activities (against MCF-7 cells) [40]. 

*C. ramiflora* was also one of the main *Cynometra* species studied, and the leaf was the most used plant part: 

A methanol extract of this medicinal plant showed significant antihyperglycemic activity [80], low anti-ulcer activity (13.9% inhibition) [35], antioxidant activity [86], and cytotoxic activity (against brine shrimp) [52].

An ethanol extract exhibited cytotoxic activity (against HeLa, T47D, and WiDr cell lines) [85,87], low antiproliferative activity (against MCF-7 cell), and weak antimicrobial activity against Escherichia coli and Bacillus subtilis [82,89]. 

An ethyl acetate fraction of a *C. ramiflora* seed methanol extract (fractioned) showed strong antioxidant and anti-lipid peroxidation activities [79], and a methanol extract of *C. ramiflora* bark showed low toxicity against mouse fibroblasts [81]. Additionally, the methanolic and an ethanol extract of *C. ramiflora* bark showed antibacterial activity (against *Vibrio cholerae*, *Salmonella typhi*, *Staphylococcus aureus*, *Escherichia coli*, *Shigella dysenteriae*, *Shigella sonnei*, *Shigella boydii*, *Shigella flexneri*, *Enterococci*, *Staphylococcus epidermis*, and *Pseudomonas aeruginosa*) [83,84]. Moreover, the bark methanol extract exhibited in vivo antinociceptive activity [83]. 

### 2.5. Toxicity

Only one study had been found concerning the toxicity of *Cynometra* medicinal plants and preparations. In this study, the authors reported that *C. ramiflora* leaf ethanolic extracts at 1000 and 1500 mg/kg BW (body weight) doses cause in vivo inflammation in the rat kidney [99]. 

No clinical toxic effects of *Cynometra* species on humans have been recorded.

## 3. Materials and Methods

This review was performed following the criteria described in the Preferred Reporting Items for Systematic Reviews and Meta-Analyses (PRISMA) statement 2020 (https://prisma-statement.org/prismastatement/flowdiagram.aspx; accessed on 1 January 2022)). 

### 3.1. Search Strategy

The scientific data were collected from PubMed, Science Direct, Web of Science, B-on, and Google Scholar, selecting all the scientific publications published between 1 January 1980 and 30 June 2022, by using keywords *Cynometra* AND medicine, *Cynometra* AND chemical compounds, *Cynometra* AND biological activity, and *Cynometra* AND toxicity. 

### 3.2. Data inclusion and Exclusion Criteria

#### 3.2.1. Inclusion Criteria

- Related to *Cynometra* genus;
- Abstract or full text in English;
- Studies on *Cynometra* species concerning medicinal importance.

#### 3.2.2. Exclusion Criteria

- Duplicate scientific publications;
- Not directly related to medicinal issues;
- Containing irrelevant or incomplete information.

## 4. Conclusion and Future Perspectives

The results of our work revealed that from the total amount of 113 species of the *Cynometra* genus, eleven (9.7%) have been reported as used in ethnomedicine, mainly for skin disease treatment. Eight species (7.1%) of this botanical genus were submitted to chemical studies and ten species (8.8%) to biological activity. The main activities evaluated were the antioxidant, antimicrobial, cytotoxic, and anti-inflammatory activities, but safety data on species of this botanical genus were almost inexistent. It has also observed that not all the species cited as used in traditional medicine, such as *C. capuronii*, *C. manii*, and *C. webberi*, were chemically or biologically studied. On the other hand, the leaf, and seed of *C. megallophylla* were documented as traditional medicines, but only the root was submitted to phytochemical studies, and no biological data have been reported concerning this species. 

The genus *Cynometra* was observed to be a botanical resource of secondary metabolites that can be related to the biological activities and the therapeutical uses described for the medicinal plants integrating it. However, to form a better conclusion about the medicinal value of each of these medicinal plants, more scientific studies concerning their safety and mode of action must be conducted, in addition to studies concerning their metabolomic, botanical, and genetic profiles, which will allow for the establishment of the much-needed quality control criteria for their better use in medicine. 

## Figures and Tables

**Table 1:** *Cynometra* species.

*Cynometra* Species		
*C. abrahamii* Du Puy & R. Rabev.	*C. craibii* Gagnep.	*C. hemitomophylla* (Donn. Sm.) Rose
*C. alexandri* C.H. Wright	*C. crassifolia* Benth.	*C. hondurensis* Dwyer
*C. americana* Vogel	*C. cubensis* A.Rich.	*C. hostmanniana* Tul.
*C. ananta* Hutch. & Dalziel	*C. cuneata* Tul.	*C. humboldtiana* Stergios
*C. ankaranensis* Du Puy & R.Rabev.	*C. cynometroides* (Merr. & L.M.Perry) Rados.	*C. inaequifolia* A.Gray
*C. aurita* R.Vig.	*C. dauphinensis* Du Puy & R. Rabev.	*C. insularis* A.C.Sm.
*C. basifoliola* (Verdc.) Rados.	*C. dongnaiensis* Pierre	*C. iripa* Kostel.
*C. bauhiniifolia* Benth.	*C. letestui* (Pellegr.) J. Léonard	*C. polyandra* Roxb.
*C. beddomei* Prain	*C. longicuspis* Ducke	*C. portoricensis* Krug & Urb.
*C. bourdillonii* Gamble	*C. longifolia* Huber	*C. psilogyne* (Harms) Rados.
*C. brachymischa* Harms	*C. longipedicellata* Harms	*C. ramiflora* L.
*C. brachyrrhachis* Harms	*C. lujae* De Wild.	*C. retusa* Britton & Rose
*C. brassii* (Merr. & L.M. Perry) Rados.	*C. lukei* Beentje	*C. rosea* (K.Schum.) Rados.
*C. browneoides* (Harms) Rados.	*C. lyallii* Baker	*C. roseiflora* W.E.Cooper
*C. capuronii* Du Puy & R.Rabev.	*C. macrocarpa* A.S.Tav.	*C. sakalava* Du Puy & R.Rabev.
*C. cauliflora* L.	*C. madagascariensis* Baill.	*C. sanagaensis* Aubrév.
*C. cebuensis* F.Seid.	*C. malaccensis* Meeuwen	*C. schefferi* (K.Schum.) Rados.
*C. cerebriformis* Rados.	*C. mannii* Oliv.	*C. schlechteri* Harms
*C. commersoniana* Baill.	*C. marginata* Benth.	*C. schottiana* Hochr.
*C. congensis* De Wild.	*C. mariettae* (Meeuwen) Rados.	*C. sessiliflora* Harms
*C. copelandii* (Elmer) Merr.	*C. marleneae A.S.Tav.*	*C. simplicifolia* Harms
*C. duckei* Dwyer	*C. mayottensis* Labat & O. Pascal	*C. sphaerocarpa* Pittier
*C. dwyeri* Rados.	*C. megalocephala* (Harms) Rados.	*C. steenisii* (Meeuwen) Rados.
*C. elmeri* Merr.	*C. megalophylla* Harms	*C. stenopetala* Dwyer
*C. engleri* Harms	*C. michelsonii* J.Léonard	*C. steyermarkii* Rados.
*C. falcata* A.Gray	*C. minor* (A.C. Sm.) Rados.	*C. suaheliensis* (Taub.) Baker f.
*C. filifera* Harms	*C. minutiflora* F.Muell.	*C. travancorica* Bedd.
*C. fissicuspis* (Pittier) Pittier	*C. mirabilis* Meeuwen	*C. trinitensis* Oliv.
*C. floretii* Labat & O.Pascal	*C. novoguineensis* Merr. & L.M.Perry	*C. tumbesiana* Rados.
*C. fortuna-tironis* (Verdc.) Rados.	*C. nyangensis* Pellegr.	*C. ulugurensis* Harms
*C. gillmanii* J.Léonard	*C. oaxacana* Brandegee	*C. vestita* (A.C.Sm.) Rados.
*C. glomerulata* Gagnep.	*C. oddonii* De Wild.	*C. vitiensis* Rados.
*C. grandiflora* A.Gray	*C. palustris* J.Léonard	*C. vogelii* Hook.f.
*C. greenwayi* Brenan	*C. parvifolia* Tul.	*C. warburgii* Harms
*C. hankei* Harms	*C. pedicellata* De Wild.	*C. webberi* Baker f.
*C. katikii* Verdc.	*C. pervilleana* Baill.	*C. yokotae* Kaneh.
*C. lenticellata* (C.T.White) Rados.	*C. phaselocarpa* (B.Heyne) J.F.Macbr./*C. spruceana* Benth.	*C. zeylanica* Kosterm.
*C. leonensis* Hutch. & Dalziel	*C. plurijuga* (Merr. & L.M.Perry) Rados.	

Adapted from: [9,10,11,12].

**Table 2:** Ethnomedicinal uses of the genus *Cynometra*.

Species	Part Used	Country	Traditional Uses	Method of Preparation	Refs.
*C. brachyrrhachis*	root	Tanzania	fungal infections	not available	[17]
*C. capuronii*	leaf	Madagascar	yellow fever	decoction	[18]
*C. cauliflora*	leaf	Indonesia	diarrhea	not available	[16]
	leaf	Malaysia	hyperlipidemia and diabetes	decoction	[15,19]
	fruit	Malaysia	loss of appetite	not available	[20]
	seed oil	India	skin diseases		
*C. hankei*	stem bark	Africa	dental pain and rheumatism		[21]
*C. iripa*	leaf, seed, stem	India	wound healing		[22]
	leaf	India	ulcers	decoction	[23]
	seed oil	India	cholera	not available	[24]
*C. manii*	stem	Nigeria	to suppress swelling in the cheeks		[25]
	bark	Nigeria	cancer		[26]
*C. megalophylla*	seed	Nigeria	fibroid treatment		[27]
	leaf	Benin	stomach infections		[28]
*C. ramiflora*	leaf, root	India	purgative, skin diseases		[29]
	whole plant	Bangladesh	skin diseases	powder	[30]
*C. spruceana*	resin	Brazil	weakness of the lungs, tuberculosis, chronic cough	not available	[31]
*C. vogelii*	stem	Nigeria	oral hygiene		[32]
*C. webberi*	root	Tanzania	skin diseases		[17]

## Data Availability

Not Applicable.

## Supplementary Materials

The following supporting information can be downloaded at: https://www.mdpi.com/article/10.3390/plants11243504/s1, Table S1: In vitro and in vivo biological studies reported from the genus *Cynometra* [15,17,19,33,34,35,40,46,47,48,49,50,51,52,53,57,58,59,60,61,62,63,64,65,66,67,68,69,70,71,72,73,74,75,76,77,78,79,80,81,82,83,84,85,86,87,88,89,90,91,92,93,94,95,96,97,98].
