# Supplementary material for: The Genus Cynometra: A Review of Ethnomedicine, Chemical, and Biological Data
Source: Plants (Basel). 2022 Dec 14;11(24):3504. doi: 10.3390/plants11243504 (PMC9781411; doi:10.3390/plants11243504)
Supplement: Supplementary file 1 [file plants-11-03504-s001.zip › plants_2065150_suplementary data S1.pdf]

Table S1. *In vitro* and *in vivo* biological studies reported from the genus *Cynometra*

Species	Part used	Extract/ compound	Biological activity	Test/Assay	Result	Ref.
C. bauhiniifolia	bark	dichloromethane	antibacterial	*In vitro* – radiometric assays	88 % inhibition of *M. tuberculosis* at 50 μg/ml	[58]
C. brachyrrhachis	root	methanol	cytotoxic	*In vitro* - MTS assay	IC_{50} values 79.2 ± 0.7 μg/ml	[17]
C. cauliflora	fruit	methanol	antioxidant	*In vitro* - beta-carotene bleaching assay	Lowest antioxidant capacity (45.95%)	[48]
	fruit	methanol	cytotoxic	*In vitro* - MTT, cell proliferation assay	CD_{50} of the extract was 0.9 μg/mL, whereas of the drug vincristine was 0.2 μg/mL after 72 hours of exposure	[59]
	leaf, fruit	aqueous, methanol	antioxidant	*In vitro* - FRAP assay and TPC assay	Higher TPC (847.31 + 26.82 mg GAE/100g) and FRAP values (19397.22 +1296.29 μM/g) in methanol extract	[60]
	leaf, fruit	water: methanol (1:3)	anti-lipase	*In vitro*	Leaf and fruit extract showed 100 % and 97.9% anti-lipase activity, respectively	[61]
	leaf	methanol	antibacterial and antioxidant	*In vitro*	The extract was more sensitive to *Staphylococcus aureus* (23.7±3.3 mm) than to *Escherichia coli*. In contrast, the extract combination with trigona honey was more sensitive to *E. coli* (23±1.9 mm) than *S. aureus* (17.6±2.6 mm). The extract and combination presented high antioxidant potentiality with IC_{50} 0.0048±0.000 mg/mL and 0.0085±0.000 mg/mL	[62]
	leaf	methanol	antidiabetic and antidiarrheal	*In vivo*- using rabbit	100 mg/mL, 250 mg/mL, and 300 mg/mL of extracts showed the ability to inhibit *α*-amylase activity by 20.6 8%, 70.24%, and 72.59%, and IC_{50} of 200.67 ± 0.53 mg/mL. Also showed antidiarrheal potentiality	[63]
	leaf	ethanol	Anti-inflammatory and antioxidant	*In vitro* – A5-LOX, hyaluronidase, xanthinoxidase enzyme inhibitory, NO production inhibitory, and DPPH assays	A5-LOX and hyaluronidase, inhibitory activities with IC_{50} values of 77.21 ± 3.14 and 25.75 ± 1.24 μg/mL respectively. At 500 g/mL extract inhibited 14.65% NO production<br><br>High xanthine oxidase inhibitory activity with 48.86% inhibition at 250 g/mL and potent antioxidant activity giving IC_{50} value 12.46 ± 0.22 μg/mL. Higher TPC and TFC were also recorded	[64]

Species	Part used	Extract/ compound	Biological activity	Test/Assay	Result	Ref.
	leaf	methanol and its fractions	*α*-glucosidase, AChE, and tyrosinase inhibitory and antioxidant	*In vitro*	Potent inhibition of all three enzymes and high antioxidant activity at $p < 0.05$	[15]
	leaf, stem, bark	aqueous	antioxidant	*In vitro*– DPPH assay	Antioxidant potentiality in the following order: young leave > matured leave > stem > bark compared with ascorbic acid. The $IC_{50\%}$ ranged from 66.36 to 258.98%	[19]
	leaf	aqueous	antioxidant and antidiabetic	*In vivo*	Significantly prevented diabetic complications associated with oxidative stress	[65]
	stem	ethyl acetate	antioxidant	*In vitro*– DPPH assay	High antioxidant activity (IC_{50} value 4.68 ± 0.035 ppm)	[53]
	fruit	hexane, chloroform, ethyl acetate, ethanol, methanol, and distilled water	antifungal and cytotoxic	*In vitro*	chloroform extract (640 μg/mL) showed significant toxicity ($P>0.001$) on the Vero cells; all extracts showed antifungal activity in different fungal species	[66]
	leaf	methanol	antibacterial	*In vitro* - Tukey-HSD test	The concentration of 100% showed the highest average inhibition zone (11.43 mm) against *Porphyromonas gingivalis*	[67]
	leaf	methanol	antibacterial	*In vitro* - broth dilution method	MIC of the extract ranged between 6.25 and 12.5 mg/ml against both *S. aureus* and MRSA. MBC values were higher than MIC, indicating that the extract is bacteriostatic at lower concentrations and bactericidal at higher concentrations	[33]
	leaf, stem	methanol	anti-AChE	*In vitro* - colorimetric 96-well microplate-based assay method	Potent anti-cholinesterase activities (> 80% inhibition) at 200μg/ml against both AChE and BchE enzymes	[68]
	leaf	ethanol, vitexin	anti-obesity and lipid-lowering effects	*In vivo*	Extract (400 and 200 mg/kg) and vitexin showed anti-obesity activity. Both doses of the extract also ($P \leq 0.05$) decreased serum triglyceride, LDL, lipase, IL-6, peptide YY, resistin levels, hyperglycemia, hyperinsulinemia, and hyperleptinemia	[69]

Species	Part used	Extract/ compound	Biological activity	Test/Assay	Result	Ref.
	fruit	aqueous	antioxidant	*In vitro*– DPPH, FRAP, and TBA (thiobarbituric acid) assays	Potential antioxidant activity and gave value 328.0 ± 9.0 μg/mL IC_{50}, 24.4 ± 2.05 μM, and 42.0 ± 0.6 AAE/gDW for DPPH, FRAP, TBA assays	[49]
	leaf	ethanol	antioxidant and alpha-Glucosidase	*In vitro* -DPPH free radical scavenging, NO scavenging, and alpha-glucosidase assays	Stronger antioxidant capability (IC_{50} value 2.88±0.05 μg/mL than the standard quercetin. High inhibitory alpha-glucosidase activity (IC_{50} value 0.90±0.02)	[46]
	fruit	ethanol	antioxidant and α-glucosidase inhibitory	*In vitro* - DPPH free radical scavenging assay, α-Glucosidase inhibitory activity assay	The extract exhibited DPPH radical scavenging activity with IC_{50} value of 11.33 ± 0.15 μg/mL and α-Glucosidase inhibitory activity with IC_{50} value of 3.01 ± 0.19 μg/mL	[47]
	fruit	methanol	antibacterial	*In vitro* - microdilution assay	No activity against Bacterial strains [MSSA (ATCC 12600 and MRSA strains (NCTC 12493 and ATCC 43300)]	[70]
	leaf	methanol	cytotoxic activity and anti-viral	*In vitro* - MTT assay; Plaque reduction assays	Cytotoxic concentration, CC_{50} of the extract was 36 mg/ mL. Potential antiviral activity against HSV-1 with effective concentration, EC_{50} = 2.14 mg/ mL and with selective index, SI value of 16.8	[71]
	leaf	methanol	cytotoxic	*In vitro* - BSLT	LC_{50} value for the extract was 196.12 ppm, and the combination of the extract with Trigona honey showed 36.6% cytotoxic activity with LC_{50} 168.2 ppm	[72]
	leaf, twig, fruit	The essential oil obtained by hydrodistillation	antioxidant, antibacterial, and cytotoxic	*In vitro* - DPPH free radical scavenging assay, Kirby Bauer assay, Broth dilution assay, MTT assay	Twig oil (IC_{50} 37.12 ± 2.84 mg/mL) exhibited better antioxidant power than the leaf (IC_{50} 207.17 ± 2.95 mg/mL) and fruit oils (IC_{50} 461.88 ± 12.61 mg/mL)<br><br>Twig oil showed activity against all microorganisms tested with inhibition zones ranging from 10.3 ± 0.4 to 29.7 ± 0.4 mm. Fruit oil exhibited antibacterial effects on *S. aureus* and MRSA, with inhibition zones of 12.7 and 11.7 mm, respectively. Twig oil decreases the proliferation of human breast cancer MCF-7 cells to 50% ($p < 0.001$)	[40]
	leaf	aqueous	antidiabetic	*In vivo* - using rat	Significant reduction in blood sugar levels at a dose of 2.500 mg per day	[73]

Species	Part used	Extract/ compound	Biological activity	Test/Assay	Result	Ref.
	fruit	aqueous	antioxidant	*In vitro* - DPPH and FRAP assay	Potential antioxidant activity giving IC_{50} value 0.47 ± 0.03 g of dry weight /mL and reducing power 25.07 ± 0.73 µmol FSE/g dry weight for DPPH and FRAP assays, respectively	[50]
	seedless fruit	methanol	antioxidant	*In vitro*- DPPH and FRAP assay	Very high antioxidant potentiality giving IC_{50} value 8.7± 0.3 in DPPH assay whereas giving low potentiality in FRAP assay by giving the value of 63.2±2.9	[51]
	leaf	ethanol	cytotoxic	*In vitro*- BSLT and MTT assay	The extract showed moderate cytotoxic activity with an LC_{50} value of 125.89 µg/mL, and a concentration of 25 µg/mL of extract inhibited the proliferation of HeLa cancer cells by 57.51%	[74]
C. cloiselii	aerial part	ethanol	antiviral	*In vitro*	Significant activity against HSV at concentrations of less than 25 µg/ml. Also showed anti- SINV activity, which was not much impressive	[75]
C. iripa	leaf	methanol, ethyl acetate, chloroform, chloroform: methanol (1:1) and hexane	antibacterial	*In vitro* - disc-assay method	Methanol, ethyl acetate, and chloroform: methanol extracts showed antibacterial activity against all tested pathogens. Chloroform extract exhibits activity against all pathogens except *Pseudomonas aeruginosa*	[76]
	leaf, stem, seed	ethanol, methanol	antibacterial	*In vitro* - cup plate diffusion method	Methanolic extracts showed high antibacterial activity against *Pseudomonas aeruginosa* as compared to ethanolic	[77]
	bark	methanol	antifungal	*In vitro* - Food poisoning method	33.70 % and 47.74% inhibition against *Alternaria alternata* and *Fusarium moniliforme*, respectively	[78]
C. madagascariensis	leaf	ethanol	antiviral	*In vitro*	Significant activity against HSV at concentrations of less than 25 µg/ml. Anti- SINV activity was not impressive	[75]

Species	Part used	Extract/ compound	Biological activity	Test/Assay	Result	Ref.
C. ramiflora	seed	ethyl acetate fraction of methanol extract	antioxidant, anti-lipid peroxidation, Cancer chemopreventive	*In vitro* - DPPH assay, thiobarbituric acid method, and quinone reductase induction method.	Strong antioxidant activity with EC_{50} value 3.33 μg/ml and strong anti-lipid peroxidation with IC_{50} value 0.8992 μg/ml. The extract did not possess cancer chemoprevention activities	[79]
	leaf	methanol	antihyperglycemic	*In vivo*	Significant antihyperglycemic activity and improvement of around 21.6% in glucose tolerance of sucrose-loaded rats	[80]
	bark	methanol	cytotoxic	*In vitro* - MTT assay	Low toxicity (IC_{50} >2.5 mg mL^{-1}) against mouse fibroblasts	[81]
	leaf	ethanol	antiproliferative	*In vitro* – MTT assay	Extract weakly inhibited the MCF-7 cell proliferation with an IC_{50} value of 317 μg/ml	[82]
	leaf	methanol	anti-ulcer	*In-vivo* - HCl/ethanol-induced ulcer assay	Low inhibitory activity (13.9 % inhibition)	[35]
	bark	methanol	antibacterial	*In vitro* - Disk diffusion method	Significant antibacterial activity against *Vibrio cholerae*, *Salmonella typhi,* and *Staphylococcus aureus*; moderate activity against *Escherichia coli*, *Shigella dysenteriae*, *S. sonnei*, *Shigella boydii*, *Shigella flexneri*, Enterococci, *Staphylococcus epidermis*	[83]
			antinociceptive	*In vivo* - acetic acid-induced writhing method using mice	Significant writhing inhibition (48.62% inhibition at the dose of 250mg/kg body weight and 63.89% inhibition at the dose of 500 mg/kg body weight)	
	stem-bark	ethanol	antibacterial	*In vitro* - solid dilution method using Mueller Hinton media	Potential antibacterial activity with an MBC value of 2% against *Staphylococcus aureus*, *Pseudomonas aeruginosa,* and *Shigella sonnei*	[84]
	leaf	ethanol	cytotoxic	*In vitro* - MTT assay	Cytotoxic effect to HeLa, T47D, and WiDr cell lines with the IC_{50} of 163.37, 533.33, and 69.66 ppm, respectively	[85]
	leaf, fruit, stem bark	methanol and its fractions	antioxidant	*In vitro* – DPPH assay	Methanolic extract, the semipolar fraction of leaf extracts, and the fraction of stem bark extract showed a potent antioxidant activity, in that order 54.44, 91.20, 79.64, and 79.59 ppm. The stem bark extract showed the highest activity (IC_{50} 41.90 ppm)	[86]

Species	Part used	Extract/ compound	Biological activity	Test/Assay	Result	Ref.
	leaf, stem bark	ethanol	cytotoxic	*In vitro* - MTT assay	Stem bark extract showed the cytotoxic effect on HeLa, T47D, and WiDr cell lines with IC_{50} values of >1000, 0.90, and 6.29 μg/mL respectively. Leaf extract exhibits a cytotoxic effect on HeLa, T47D, and WiDr cell lines with IC_{50} values of 1,92, 6.37, and 0,41 μg/mL respectively	[87]
	leaf	fractions of ethanol extracts	cytotoxic	*In vitro* - MTT assay	The highest cytotoxic activity was showed by a polar fraction with IC_{50} of 260.0171 g/mL, IC_{50} non-polar fraction of 294.7592 g/mL, and the lowest activity by the semipolar fraction with IC_{50} 318.6368 mg/mL against T47D cells	[34]
	leaf	80% methanol	antioxidant	*In vitro* – DPPH assay	Low antioxidant activity with EC_{50} value 250.00 μg/ml	[88]
	leaf, stem	methanol, chloroform	antioxidant, antimicrobial, and cytotoxic	*In vitro* - DPPH, disc diffusion method, brine shrimp lethality bioassay	Stem methanolic extract showed the highest antioxidant activity (IC_{50} 31.62 μg/mL^{-1}). The chloroform extract of the stem exhibited moderate antimicrobial activity against several bacterial strains (MIC values 62.5 to 500μg.mL^{-1}). The methanolic stem and leaf extracts demonstrated strong lethality in preliminary cytotoxicity assay where LC_{50} values were 1.596 and 4.613 μg/mL^{-1} respectively	[52]
	leaf	ethanol	anti-bacterial	*In vitro* – microdilution technique and a disc diffusion method	Less antimicrobial potentiality against *Escherichia coli* and *Bacillus subtilis* (MIC value 250 and 125 μg/ml, respectively)	[89]
	leaf	not indicated	antiviral	*In vitro* - on Huh7it-1 cell- Focus assay, MTT assay	The CC_{50} value and IC_{50} values were 125/mL and 20.1/mL, respectively, for the dengue virus	[90]
	leaf	$Feso_4$. $7h_2o$: aqueous extract (1:2)	antibacterial	*In vitro* - Kirby-Bauer diffusion assay	The synthesized iron oxide nanoparticles exhibited effective inhibition against *Escherichia coli* and *Staphylococcus epidermis*	[91]
	leaf	not indicated	antiviral	*In vitro* -on Huh 7.5 cell -foci-forming immunoassay	Potentiality as anti-dengue. Administration of extract at 1,25; 2,5 ;5; 10 and 20 μg/ml resulting 36,06 %, 45,96 %, 47,35%, 55,94%, 62,70% inhibition towards Dengue virus respectively	[92]

Species	Part used	Extract/ compound	Biological activity	Test/Assay	Result	Ref.
	leaf	methanol	xanthine oxidase inhibitor	*In vitro*	The extract inhibited the action of xanthine oxidase	[93]
C. spruceana	aerial part	methanol: dichloromethane (1:1)	cytotoxic	*In vitro* - cell culture sulforhodamine B method	Active against human adenocarcinoma colon cancer cell line KM-12. (Lethality 18.08%)	[94]
	aerial part	methanol: dichloromethane (1:1)	cytotoxic	*In vitro* - cell culture sulforhodamine B method	The extract showed less activity against the KB-ADL#12 cell line	[95]
C. travancorica	leaf	methanol	antioxidant and anti-inflammatory	*In vitro* and *in vivo*- superoxide scavenging, hydroxyl radical scavenging, and inhibition of lipid peroxidation assays, carrageenan, and formalin-induced paw edema assay	Significant antioxidant activity. Oral administration of extract significantly and dose-dependently reduced the paw edema	[96]
	bark	methanol	antioxidant	*In vivo* - using Swiss albino mice	Catalase activity was found to be significantly increased in mice treated with 200 and 400 mg/Kg while the percentage increase of catalase activity was 15.92 and 49.19% ($P < 0.001$) and the percentage increase of glutathione was found to be 9.75 and 56.36%	[97]
	stem bark	methanol	antioxidant	*In vitro* - DPPH, superoxide radical scavenging *In vivo*-NaF induced oxidative stress using mice	The extract increased catalase (46.6%), superoxide dismutase (53.8%) activities, and GSH level (48.1%) against NaF-induced decline in the liver tissue of mice	[98]
C. vogelii	leaf	essential oil	antifungal	*In vitro*	Significant antifungal activity against *Cryptococcus neoformans* (MIC = 80 μg/mL) but no antifungal activity against *Aspergillus niger* or *Candida albicans*	[57]

TFC- total flavonoid content; TPC- total phenolic content, DPPH- 2,2-diphenyl-1-picrylhydrazyl; FRAP- ferric reducing antioxidant power; NO- nitric oxide; NaF- Sodium fluoride; SINV- sindbis virus; HSV- herpes simplex virus; LC_{50}- lethal concentration 50%, A5- LOX-Arachidonate-5-lipoxygenase; IC_{50}- half-maximal inhibitory concentration; EC_{50}-half maximal effective concentration; CD_{50}- half maximal cytotoxic dose; CC_{50}- half maximal cytotoxic concentration; FSE- ferrous sulphate equivalents; BSLT- brine shrimp lethality test; MTT- microculture tetrazolium technique; MIC -minimum inhibitory concentrations; MBC-minimum bactericidal concentrations; MSSA- methicillin-sensitive *Staphylococcus aureus*; MRSA - Methicillin-resistant Staphylococcus aureus; GAE- gallic acid equivalent; TBA- thiobarbituric acid, BChE- butyrylcholinesterase; AChE- acetylcholinesterase
